# Expanding treatment options for syphilis during pregnancy: a review of alternatives to penicillin

**DOI:** 10.3389/fgwh.2026.1879243

**Published:** 2026-08-27

**Authors:** Claire L. Townsend, Lynne Mofenson, Sami Gottlieb, Remco P. H. Peters, Oriol Mitjà, Clara Pérez-Mañá, Elaine J. Abrams, Sinead Delany-Moretlwe, Shahin Lockman, Catriona Waitt, Kathryn Miele, Melanie M. Taylor, Françoise Renaud

**Affiliations:** 1 Department for HIV, Tuberculosis, Hepatitis and Sexually Transmitted Infections, World Health Organization, Geneva, Switzerland; 2 Elizabeth Glaser Pediatric AIDS Foundation, Washington, DC, United States; 3 Department of Sexual, Reproductive, Maternal, Child, Adolescent Health and Ageing, World Health Organization, Geneva, Switzerland; 4 STI and Skin NTDs Section, University Hospital Germans Trias I Pujol, Badalona, Spain; 5 Department of Medicine, Universitat Autònoma de Barcelona, Bellaterra (Cerdanyola del Vallés), Spain; 6 Department of Infectious Diseases, Universitat de Vic-Universitat Central de Catalunya, Vic, Spain; 7 Department of Pharmacology, Universitat Autònoma de Barcelona, Bellaterra (Cerdanyola del Vallés), Spain; 8 ICAP at Columbia University and the Department of Epidemiology, Mailman School of Public Health, Columbia University, New York, NY, United States; 9 Department of Pediatrics, Vagelos College of Physicians & Surgeons, Columbia University, New York, NY, United States; 10 Wits RHI, University of the Witwatersrand, Johannesburg, South Africa; 11 Department of Immunology and Infectious Diseases, Harvard T.H. Chan School of Public Health, Boston, MA, United States; 12 Division of Infectious Diseases, Brigham & Women’s Hospital, Boston, MA, United States; 13 Department of Women’s and Children’s Health, University of Liverpool, Liverpool, United Kingdom; 14 Infectious Diseases Institute, Makerere University College of Health Sciences, Kampala, Uganda; 15 Division of STD Prevention, National Center for HIV, Viral Hepatitis, STD, and TB Prevention, Centers for Disease Control and Prevention, Atlanta, GA, United States; 16 Division of HIV Prevention, National Center for HIV, Viral Hepatitis, STD, and TB Prevention, Centers for Disease Control and Prevention, Atlanta, GA, United States

**Keywords:** pregnancy, review, safety, syphilis, therapeutics, treatment

## Abstract

Syphilis infection during pregnancy leads to around 390,000 adverse birth outcomes each year and is an important cause of preventable stillbirth. Although benzathine penicillin G is safe and effective during pregnancy for treating maternal and fetal infection, there are frequent global shortages and limited options for people with penicillin allergy. We conducted a review of potential alternatives to penicillin for treating syphilis during pregnancy when penicillin is not available or cannot be used, including ceftriaxone, amoxicillin, doxycycline, cefixime, and linezolid. Despite some efficacy data on their use in non-pregnant adults with syphilis, limited data were available on placental transfer, pharmacokinetics and dosing, efficacy or effectiveness, and safety in pregnancy. Given its favourable preclinical reproductive toxicity data, efficacy data in non-pregnant adults with syphilis, and safety during pregnancy when used for other indications, ceftriaxone could potentially be considered as an alternative treatment for pregnant women with syphilis in cases where penicillin cannot be used, as currently recommended by the World Health Organization. However, evidence about optimal dosing, feasibility, and efficacy/effectiveness for treating syphilis during pregnancy is needed. Several planned and ongoing clinical trials will provide additional data to inform the use of cefixime, amoxicillin, and linezolid in pregnant women with syphilis. Going forward, evaluation of new and existing medications for treating syphilis and preventing congenital syphilis should include a pregnancy investigation plan. Where possible, efforts should be made to ensure that high-quality pregnancy safety and efficacy or effectiveness data on drug candidates are generated in carefully designed studies to ensure that pregnant women and their infants are able to benefit from the full range of treatment options.

## 1 Introduction

Syphilis is a sexually transmitted infection of major public health concern, with potentially severe and lifelong consequences for untreated adults and infants who acquire infection and are not adequately treated *in utero*. The World Health Organization (WHO) has called for the elimination of congenital syphilis (infection acquired during pregnancy or at birth) as part of its triple elimination strategy for HIV, syphilis, and hepatitis B virus (1). However, in 2022, there were an estimated 700,000 congenital syphilis cases and 390,000 syphilis-associated adverse birth outcomes globally (2–4). Concerningly, syphilis rates have been rising in many regions in recent years, including in the United States, Europe, and Asia (5).

Syphilis can be identified and successfully treated during pregnancy. However, when left untreated, or treated late or inadequately, syphilis can result in multiple adverse birth outcomes including stillbirth, miscarriage, neonatal death, preterm birth or low birth weight, and congenital infection in the infant (6, 7). Syphilis accounts for nearly 8% of all stillbirths globally, making it a key preventable cause (8). Congenital infection is associated with a range of clinical manifestations in the infant affecting multiple organ systems (9). The risk of congenital syphilis varies according to stage of maternal infection. Untreated early infection leads to congenital syphilis in 50%–70% of infants, while infection acquired more than a year before pregnancy is associated with a lower risk of congenital infection, around 15% (10).

The recommended treatment for early (primary, secondary and early latent) syphilis during pregnancy is a single intramuscular injection of 2.4 million units (MU) of benzathine penicillin G (BPG) (11–13). Most cases of syphilis infection during pregnancy are asymptomatic and are classified as late latent infection—contracted more than 1–2 years previously—or latent infection of unknown duration (14). For these latent infections, three doses of BPG given at weekly intervals are recommended, although evidence to support this approach is limited. If BPG is not available, WHO recommends 1.2 MU of procaine penicillin given intramuscularly (IM) once daily for 10 or 20 days for early or late syphilis, respectively (11). For pregnant women with penicillin allergy, desensitization followed by treatment with BPG is typically recommended (11–13, 15, 16).

Although BPG is an effective and well-tolerated treatment for syphilis during pregnancy, there have been recurring global shortages and national stockouts of BPG in recent years (17, 18). Furthermore, there are acceptability challenges linked to IM administration, and additional options are needed for pregnant women with penicillin allergy (17). Therefore, there is a need for acceptable, safe and effective treatment alternatives for use in pregnant women with syphilis when BPG is not available or cannot be used (15, 19). The identification of oral alternatives to BPG for treating syphilis during pregnancy has been highlighted by WHO as a research priority (20). Potential alternatives to penicillin include ceftriaxone, doxycycline, and amoxicillin. Linezolid and cefixime have emerged more recently as possible options (19, 21). Alternatives need not only to be safe and effective for treating maternal syphilis but also to have proven effectiveness in treating fetal infection and preventing congenital infection. The aims of this review were (1) to describe the landscape of potential alternatives to penicillin for treating syphilis during pregnancy in cases where BPG is not available or cannot be used, and (2) to identify evidence gaps and future research needs to inform global guidance on the use of alternative agents for treating syphilis during pregnancy.

## 2 Methods

A narrative literature review on potential alternative agents to penicillin for the treatment of syphilis during pregnancy was undertaken. As the main scope of this review was to provide an overview of the therapeutic landscape, the initial searches were conducted in PubMed only, with additional relevant publications identified by screening reference lists of selected papers. Articles in English or French published anytime up to 10 April 2025 were included, using search terms relating to syphilis [“syphilis” [MeSH Terms], “syphili*”, “treponema pallidum” [MeSH Terms]], pregnancy [“pregnancy” (MeSH Terms), “pregnan*”, “antenatal*”, “prenatal*”, “maternal*”] and specific drug names [“cefixime”, “linezolid”, “ceftriaxone”, “amoxicillin” (or “amoxycillin”), and “doxycycline”] (Search 1). Additional searches were run in PubMed to identify reviews, systematic reviews and meta-analyses published in the last 10 years (10 April 2015 to 10 April 2025) on treatment for syphilis during pregnancy, using search terms relating to syphilis and pregnancy (as above) along with generic treatment/antimicrobial terms [“anti-bacterial agents” (MeSH Terms), “treat*”] (Search 2). Articles reporting on or discussing the use of any of the five above-mentioned drugs for treatment of syphilis during pregnancy were deemed eligible. Grey literature searches included relevant international guidelines, drug product labels, and abstract books from the 2024 International Union against Sexually Transmitted Infections (IUSTI) World Congress, the 2025 Sexually Transmitted Infections (STI) & HIV World Congress, and the 2025 IUSTI Europe Congress.

The following clinical trial registries were searched for planned and ongoing studies using the terms “syphilis” and specific drug names (“cefixime”, “linezolid”, “ceftriaxone”, “amoxicillin”, and “doxycycline”): ClinicalTrials.gov; WHO International Clinical Trials Registry Platform (http://apps.who.int/trialsearch/); EudraCT (https://www.clinicaltrialsregister.eu/ctr-search/search); and the Chinese Clinical Trial Registry (https://www.chictr.org.cn/). Trials investigating treatment for neurosyphilis only were excluded.

**Figure 1:**
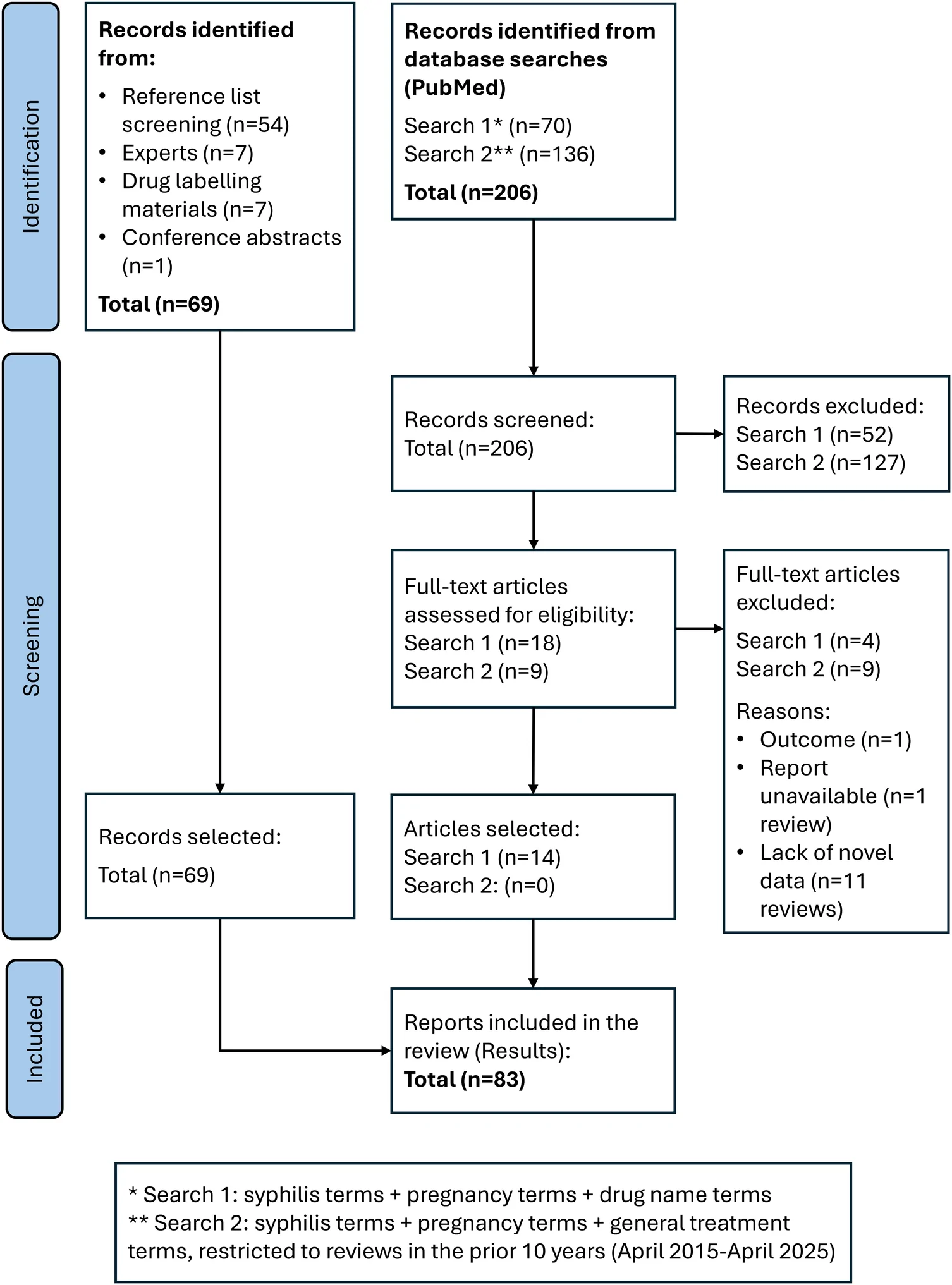
PRISMA flow diagram showing the inclusion and exclusion of studies at each stage of the review.

All abstracts were screened for eligibility in Microsoft Excel by a single reviewer (CT), and full text articles deemed relevant were retrieved and assessed for inclusion by the same reviewer. A PRISMA flow diagram showing the number of articles identified, screened, and included is shown in Figure 1. Data from the source documents (articles retrieved through the initial searches) on preclinical studies, pharmacokinetics (PK) and dosing, and efficacy/effectiveness and safety in pregnant women and non-pregnant adults were initially synthesized using Google NotebookLM, an artificial intelligence (AI) research tool powered by Gemini, version 2.0. The tool assisted in identifying key data sources and themes for each drug, and original sources were then independently reviewed and verified for accuracy by the lead authors.

Syphilis disease stages were defined as early syphilis, consisting of primary, secondary and early latent syphilis (asymptomatic, of less than 1–2 years' duration), and late syphilis, consisting of late latent syphilis (of more than 1–2 years' duration) and tertiary syphilis (11). Serological cure was defined as a four-fold reduction in rapid plasma reagin (RPR) titre, unless otherwise specified. Members of the WHO HIV, Hepatitis and STIs Pregnancy and Breastfeeding Therapeutics Working Group (PTWG) reviewed the summary results for accuracy and completeness and provided information on upcoming funded studies that were not included in trial registries.

## 3 Results

### 3.1 Ceftriaxone

Ceftriaxone is a third-generation cephalosporin effective against syphilis (Table 1) (22). Preclinical developmental and reproductive toxicity studies in mice, rats, and primates have shown no evidence of embryotoxicity, fetotoxicity, or teratogenicity (Table 2). While adequate and well-controlled safety studies in pregnant women with syphilis have not been conducted, the drug is considered by the US Food and Drug Administration (FDA) as safe for use in pregnancy (23) and it is used for a range of other conditions, including Lyme disease, urinary tract infections, and gonorrhoea during pregnancy (24–26).

**Table 1:** Drug characteristics (class, formulation, dosing, efficacy against *Treponema pallidum*, and placental transfer) and WHO recommendations on alternative treatments for syphilis during pregnancy.

Drug	Antibiotic class	Formulation	Dosing for syphilis treatment (pregnant and non-pregnant adults)	WHO syphilis treatment recommendation (11)	Efficacy against *T. pallidum* (*in vitro*)	Placental transfer
Ceftriaxone	Cephalosporin	IM injection	In non-pregnant adults: 1 g IM injection daily for 10–14 days. Dosing in pregnancy not yet established.	Alternative treatment for pregnant women in rare cases that BPG is not available (and providing there is enhanced follow-up)	Yes, MIC 0.0007–0.0025 mg/L (28, 29).	Yes, cord blood levels 97% of maternal levels (32)
Cefixime	Cephalosporin	Oral	Dose of 400 mg twice daily for 10 days currently being tested in non-pregnant adults.	Not currently recommended for treatment of syphilis in pregnant or non-pregnant adults.	Yes, MIC 0.0313 mg/L (29)	Likely—amniotic fluid levels ∼38% of maternal serum levels in small study (46).
Amoxicillin (±probenecid)	Penicillin derivative	Oral	Dose in pregnancy is 1,500 mg/day (500 mg orally every 8 h) for 2–12 weeks depending on syphilis disease stage (Japanese guidelines). Specific dosing for syphilis treatment in pregnancy not yet established.	Not currently recommended for treatment of syphilis in pregnant or non-pregnant adults.	Yes, MIC 0.02 mg/L (29)	Yes, cord blood levels ∼18–30% of maternal levels (56, 57).
Doxycycline	Tetracycline	Oral	In non-pregnant adults: 100 mg orally twice daily (or 200 mg once daily) for 14 to 28 days, depending on disease stage. Dosing in pregnancy not yet established.	Alternative treatment for non-pregnant adults with penicillin allergy; not recommended in pregnancy	Yes, MIC 0.10 mg/L (29)	Tetracyclines shown to cross the placenta, no studies on doxycycline.
Linezolid	Oxazolidinone	Oral	In non-pregnant adults: 600–1,200 mg daily for 10–28 days; no dosing adjustment in pregnancy but data limited.	Not currently recommended for treatment of syphilis in pregnant or non-pregnant adults. Cautious use in pregnancy for drug-resistant TB, with monitoring (88).	Yes, MIC 0.5 mg/L (29)	Expected to cross the placenta; single case report (94).

CNS, central nervous system; IM, intramuscular; MIC, minimum inhibitory concentration.

**Table 2:** Summary of available preclinical data and evidence on pharmacokinetics, efficacy and safety of alternative treatments for syphilis during pregnancy.

Drug	Pre-clinical development + reproductive toxicity studies (from product labels)	Clinical efficacy against syphilis in adults	Pharmacokinetic (PK)/ pharmacodynamic studies in pregnant women	Dedicated pregnancy safety studies (assessing pregnancy and birth outcomes)	Observational studies in pregnant women with syphilis or other indications (short and long-term safety)	Considerations
Ceftriaxone	No evidence of embryotoxicity, fetotoxicity or teratogenicity at doses up to 20× human equivalent dose (rats and mice).	Yes, serological cure rate of 92% at 12 months (27).	Yes, similar in pregnant and non-pregnant women, but optimal dose and duration of ceftriaxone during pregnancy for syphilis not clearly defined (15, 33).	None available.	Case reports (*n* = 14 pregnancies) and one observational study (*n* = 79) in women with syphilis (15, 39).	Requires daily IM injections or IV administration for 10–14 days. Regimen adherence may be problematic.
Cefixime	No evidence of teratogenicity or embryotoxicity at doses up to 40× human dose; no effects on fertility or reproductive performance at doses up to 25× human dose.	Yes, 87% response rate vs 93% in penicillin arm in pilot RCT (45). RCT preliminary results: 90% cure rate for primary/secondary syphilis (48).	Data limited to one case report.	None available.	None for syphilis; Limited observational data on use for other indications in pregnancy.	
Amoxicillin (±probenecid)	No evidence of harm to fetus at doses 3–6× human dose.	Yes, 90–95% cure rate based on a pilot RCT and observational studies (21, 27, 51).	Renal excretion estimated to be higher and half-life shorter (by 19–31%) in pregnant versus non-pregnant adults (13, 43). Modelling suggests need for more frequent dosing in later pregnancy (13).	None available.	Case reports and one retrospective study in women with syphilis (total *n* = 85) (15). Cases of congenital syphilis reported in women with late syphilis (*n* = 15/41) (55). Reviews of observational studies do not raise any clear safety issues in pregnancy.	Concerns regarding prevention of congenital infection in women with late syphilis require further investigation.
Doxycycline	Tetracyclines associated with embryotoxicity; no evidence of teratogenicity with doxycycline; skeletal anomalies and decreased fetal weight observed at high doses (>17× human dose).	Yes, similar response to BPG in observational studies (27). No clinical trials to date.	None available.	None available.	Single case report on use of doxycycline in a woman with syphilis (79). Observational studies of doxycycline in pregnancy for other indications show no evidence of teratogenicity, but lack of good quality studies (72, 74, 80).	Safety in pregnancy requires further investigation given tetracycline class toxicity; unclear if this extends to doxycycline.
Linezolid	No teratogenic effects, but other adverse effects noted at human equivalent doses (decreased survival and fertility, delayed auditory response and locomotor reflexes). Total litter loss reported in 8/25 mice given a dose approx. 1.8 to 3-fold higher than human dose.	600 mg once daily for 5 days showed only 70% response (95); a regimen of 600 mg twice daily for 10 days is currently being investigated (96).	No dosing adjustment required in pregnancy, but data limited to one case report (97). PK studies needed.	None available.	Case reports—31 cases of pregnant women with linezolid exposure for treatment of tuberculosis (19).	High efficacy against T. pallidum in *in vitro* and *in vivo* models—duration and dosing to be determined.

RCT, randomized controlled trial.

Some national and international guidelines propose ceftriaxone, administered IM or intravenously (IV) daily for 10–14 days, as an alternative treatment for early syphilis during pregnancy in cases of penicillin allergy without the possibility of penicillin desensitization, or when the recommended penicillin regimens are unavailable (Table 1) (11, 12). The optimal duration of ceftriaxone treatment for late syphilis is not well established. Although cross-reactivity between cephalosporins and penicillin have been reported in people with penicillin allergy, such reactions rarely occur with third generation cephalosporins such as ceftriaxone and cefixime (13). Ceftriaxone is less convenient than BPG because of the need for daily parenteral dosing over 10–14 days, which is challenging to implement in many settings (11, 12, 27).

#### 3.1.1 Preclinical data

*In vitro* and animal studies have demonstrated activity of ceftriaxone against *Treponema pallidum,* the bacterium that causes syphilis (Table 1). The minimum inhibitory concentration (MIC) of ceftriaxone for *T. pallidum* has been estimated at 0.0007 mg/L (28) and 0.0025 mg/L (29). The minimum bactericidal concentration (MBC) is also low, approximately 0.002 µg/mL, similar to penicillin (28).

Animal studies in rats and mice showed no evidence of embryotoxicity, fetotoxicity, or teratogenicity at doses up to 20 times the human equivalent dose (Table 2). In primates, no embryotoxicity or teratogenicity was demonstrated at a dose approximately three times the human dose (23). In rats, no adverse effects on various reproductive parameters during gestation and lactation were noted, including postnatal growth, functional behaviour, and reproductive ability of the offspring, at doses of 586 mg/kg/day or less (23).

#### 3.1.2 Pharmacokinetics and dosing

Ceftriaxone is administered as an IV or IM injection of 1 g of ceftriaxone daily for 10–14 days for primary or secondary syphilis (13). Maternal serum levels achieved after a 1 g parenteral dose have been reported to be adequate (median 83 µg/mL; peak 138 µg/mL) (30, 31). Cord blood concentrations were measured to be 97% of maternal serum concentrations in a study of 31 women with premature rupture of membranes (32). In a review published in 2025, ceftriaxone PK was reported to be similar in pregnant and non-pregnant adults, although this was based on limited data (15, 33). There is some evidence of slower elimination in pregnant vs. non-pregnant adults based on a small study of pregnant women undergoing Caesarean section, but results were not conclusive (34). A physiologic-based population PK model predicted a significant decrease in maternal and fetal ceftriaxone exposure in the third trimester compared with outside of and earlier in pregnancy for both a 1 g and 2 g dosing approach (35). In the absence of clear data suggesting the need for dosing adjustment, pregnant women with primary or secondary syphilis can likely receive the same dose and regimen as non-pregnant adults (1 g daily, IM or IV, for 10–14 days), but confirmation is needed (15).

#### 3.1.3 Efficacy/effectiveness data in non-pregnant adults with syphilis

The clinical efficacy of ceftriaxone for treating syphilis has been demonstrated in a randomized non-inferiority trial in 301 non-pregnant adults in China [112 on ceftriaxone (64 women), 118 on BPG]. The trial reported a serological cure rate of 92% at 12 months among those on ceftriaxone and 81% in those on BPG (Table 2) (36). In addition, a meta-analysis of seven earlier randomized controlled trials, including a total of 281 non-pregnant participants (159 on ceftriaxone), showed no significant difference in the 6-month or 12-month response rate with ceftriaxone compared with penicillin (the number of men and women included was not reported) (22).

#### 3.1.4 Efficacy/effectiveness and safety data in pregnant women

##### 3.1.4.1 Treatment for syphilis

Clinical data on the use of ceftriaxone in pregnant women with syphilis are limited and mostly consist of case reports (Table 2). These are summarised in a 2025 review (15): among 14 individual case reports with varied regimens (30, 31, 37, 38), there were no cases of congenital syphilis, but follow-up was variable and limited (in some cases <6 months) (15). In addition, a retrospective case review of 79 pregnant participants treated with 250–500 mg of ceftriaxone IM for 10–14 days (depending on stage) was reported from Latvia (39). Serological response [two-fold reduction in *Treponema Pallidum* Hemagglutination test (TPHA) antibody titre after 12 months of treatment] was reported in all 26 women with secondary syphilis and in 51/53 women with early latent syphilis, with no adverse reactions and no clinical relapses; however, there were a number of limitations to the study, including limited details on regimens, use of lower than recommended dosing, nonstandard definitions of clinical response, and lack of information on birth outcomes and congenital syphilis (15).

##### 3.1.4.2 Treatment for other conditions

As mentioned above, ceftriaxone is used for a range of other conditions during pregnancy and is considered safe for pregnant women (23, 25, 26). While the risk is likely rare, some parties suggest cautious utilization of ceftriaxone in neonates due to the potential for displacement of bilirubin from albumin and concerns around neonatal hyperbilirubinemia or kernicterus (23, 40).

### 3.2 Cefixime

Cefixime is an oral third-generation cephalosporin. Reproductive toxicity studies in animals have not demonstrated a risk to the fetus, and cefixime has been used during pregnancy for a variety of other conditions such as uncomplicated urinary tract infections and gonorrhoea (25, 41–43). However, adequate and well-controlled safety studies in pregnant women have not been conducted (44). Given that there are limited data on the use of cefixime for the treatment of syphilis in non-pregnant adults, and no studies on syphilis during pregnancy, cefixime is not currently recommended to treat pregnant or non-pregnant adults with syphilis (Table 1) (12, 13, 16).

#### 3.2.1 Preclinical data

Cefixime has been shown to have *in vitro* antibacterial activity against *T. pallidum*, with significant inhibition of bacterial growth at an MIC of 0.0313 mg/L (29). Cefixime is not considered teratogenic; reproduction studies of cefixime in mice and rats at doses up to 40 times the human dose do not suggest any risk of harm to the fetus (Table 2) (44). In rats, cefixime given at doses up to 25 times the adult therapeutic dose did not affect fertility or reproductive performance (44).

#### 3.2.2 Pharmacokinetics and dosing

The recommended dose of cefixime for treating infections such as uncomplicated urinary tract infections, otitis media, pharyngitis, and tonsillitis is 400 mg once daily (44). For treatment of syphilis, a dose of 400 mg every 12 h has been estimated to be required to achieve sufficient concentrations. In ongoing clinical trials in non-pregnant adults with early syphilis, cefixime is administered orally at a dose of 400 mg twice daily for 10 days (41, 45).

Cefixime has been shown to cross the placenta and was detected in amniotic fluid in six pregnant women, at a level of 0.85 ± 0.42 *μ*g/mL, about 38% of maternal serum concentrations (2.59 ± 1.10 μg/mL) (Table 1) (46). These levels are higher than the MIC for *T. pallidum* (41), and cefixime could therefore be considered suitable for treating infections involving the membranes and placenta (46). To date there have been no dedicated PK studies of cefixime in pregnant women, but a study to assess cefixime PK in healthy pregnant and non-pregnant women in Papua New Guinea is planned to start in 2026 (Table 3). In addition, pregnancy PK data will be collected in a randomized non-inferiority trial of cefixime vs. BPG during pregnancy (Table 3).

**Table 3:** Summary of research gaps relating to alternative treatments for syphilis during pregnancy and corresponding ongoing or funded studies aiming to address these gaps.

Drug	Research gap	Study type	Planned/ongoing studies identified through trial registry searches or PTWG members
Ceftriaxone	Efficacy in non-pregnant adults with syphilis		- Comparative study of injection benzathine penicillin and injection ceftriaxone in the management of early syphilis in non-pregnant adults (CTRI/2024/07/071014; PI: Jino B G Briswin; 2024-)
	Optimal dose and duration in pregnancy	Pregnancy PK/dosing	- IMPAACT 2044: phase 4, non-randomized, opportunistic study of PK and safety of IV or IM ceftriaxone versus BPG in pregnant women with syphilis, or ceftriaxone for indications other than syphilis (NCT07207876; PI: Lisa Levy; 2025–2027)
	Efficacy and safety in pregnant women with syphilis (including for congenital syphilis)	RCT or robust observational study	
	Longer-term safety in pregnancy	Observational studies/surveillance	
Cefixime	Efficacy in non-pregnant adults with syphilis	RCT	- Phase 1/2 study of cefixime vs BPG in non-pregnant women in Brazil (CeBra) (NCT03752112; PI: Melanie Taylor; 2019–2025 41) - Phase 3 non-inferiority trial of cefixime vs BPG in USA and Peru (NCT04958122; PI: Jeffrey D Klausner; 2020–2027)—pregnant women excluded
	Optimal dose and duration in pregnancy	Pregnancy PK/dosing	- PK study in pregnancy, Papua New Guinea (ACTRN12625001176459; PI: Brioni Moore; 2025–2027)* 1. Study 1: Single-dose PK study in pregnant and non-pregnant women (*n* = 64; ACTRN12625001180404) 2. Study 2: Multiple-dose PK study: dose finding and tolerability assessment of 14-day course in pregnancy (*n* = 32; ACTRN12625001175460) 3. Study 3: Transplacental transfer study: single dose in labour with evaluation of cord blood, and maternal and neonatal specimens (*n* = 58; ACTRN12625001176459) - PK sub-study, RCT of cefixime versus BPG in pregnancy (PI: Oriol Mitjà; 2026-; not yet registered)*
	Efficacy and safety in pregnant women with syphilis (including for congenital syphilis)	RCT or robust observational study	- RCT of cefixime versus BPG in pregnancy (PI: Oriol Mitjà; 2026-; not yet registered)*
	Longer-term safety in pregnancy	Observational studies/surveillance	
Amoxicillin	Efficacy in non-pregnant adults with syphilis	RCT	- Phase 3 trial of oral amoxicillin vs BPG for treatment of congenital syphilis, Argentina (AMOXSYP; NCT06877351; 2024–2027) - Phase 4 RCT on efficacy of amoxicillin vs BPG for early syphilis in Japan (JPRN-jRCT1031210615/JPRN-UMIN000046856; PI: Naokatsu Ando; 2022–2025)
	Optimal dose and duration in pregnancy	Pregnancy PK/dosing	- Phase 1 Study of the Pharmacokinetics of Amoxicillin in Pregnancy, USA (NCT05309928; PI: Jodie Dionne-Odom, 2023–2025)
	Efficacy and safety in pregnant women with syphilis (including for congenital syphilis)	Observational studies/surveillance	
Doxycycline	Efficacy in non-pregnant adults with syphilis	RCT	- Phase 2 trial of doxycycline (200 mg once-daily, 14d) for early syphilis, Washington DC, USA (NCT06683638; PI: Travis Hunt, 2025-) - Phase 3 trial of doxycycline (100 mg, 14d) vs BPG for early syphilis in France (SY-DOXY) (NCT04838717; PI: Nicolas Dupin; 2021–2025) - RCT of doxycycline vs BPG in non-pregnant adults with early syphilis in India (CTRI/2023/05/052508, PI: Vasireddy Teja; registered May 2023).
	Optimal dose and duration in pregnancy	Pregnancy PK/dosing	
	Placental transfer and efficacy for preventing congenital syphilis	*In vivo* or *in vitro* placental studies	
	Safety in pregnancy (any indication)	Systematic review	
	Efficacy and safety in pregnant women with syphilis [primarily birth outcomes (miscarriage), congenital abnormalities, teeth staining]	RCT or robust observational study	
Linezolid	Efficacy in non-pregnant adults with syphilis; optimal dose and duration of treatment	RCT	- Phase 2 pilot study of linezolid for syphilis, USA, Peru (NCT05548426; PI: Jeffrey D Klausner; 2023–2025 96)—pregnant women excluded - Phase 3 study of linezolid for syphilis (Trep-AB-1200), Spain (NCT05069974; PI: Oriol Mitjà; 2021–2025 91)—pregnant women excluded
	Optimal dose and duration in pregnancy	Pregnancy PK/dosing	
	Efficacy and safety in pregnant women with syphilis (including for congenital syphilis)	RCT or robust observational study	
	Longer-term safety in pregnancy	Observational studies/surveillance	
Alternative penicillin-based approaches	Efficacy in non-pregnant adults with syphilis	RCT	- Non-randomized Phase 2 trial of single dose subcutaneous penicillin vs 3 doses of IM BPG in non-pregnant adults (ACTRN12625000210471; PI: Laurens Manning, 2025-)*
	Efficacy and safety in pregnant women with syphilis (including for congenital syphilis)	Pregnancy PK, RCT	- Trial of single dose subcutaneous penicillin (7.2 MU) vs 3 doses of IM BPG in pregnancy (planned; PI: Michael Marks; 2026-; not yet registered)*

* Studies funded but not yet registered (or registered subsequent to registry searches), identified through PTWG members.

BPG, benzathine penicillin G; CDC, United States Centers for Disease Control and Prevention; IM, intramuscular; IV, intravenous; PI, principal investigator; PK, pharmacokinetic(s); PTWG, World Health Organization HIV, Hepatitis and STIs Pregnancy and Breastfeeding Therapeutics Working Group (PTWG); RCT, randomized controlled trial; MU, million units; USA, United States of America.

#### 3.2.3 Efficacy/effectiveness data in non-pregnant adults with syphilis

A pilot non-comparative study evaluated oral cefixime 400 mg twice a day for 10 days in 58 non-pregnant adults with early syphilis (nearly all participants overall, and all those in the per-protocol arm were male) (47). The per-protocol treatment response was 87% (13/15) in the cefixime arm, and 93% (14/15) in the penicillin arm (intention-to-treat response: 56% and 81%, respectively) (Table 2) (45). In light of these results, a larger non-inferiority RCT was launched, with preliminary results suggesting a 90% success rate at 6 months among participants with early syphilis (51/57; compared with 96%, 48/50, for penicillin; participants were mostly men who have sex with men) (48).

Another RCT in 61 participants with early syphilis (58 male) reported serological response [four-fold reduction in venereal disease research laboratory (VDRL) test titre] at 3 months in 73% (22/30) of those who received oral cefixime (400 mg twice daily) and 87% (27/31) of those in the BPG arm; at 12 months, serological response was achieved in 93% (28/30) of participants in the cefixime arm and 97% (30/31) of those on BPG (49). An ongoing randomized controlled trial (NCT03752112) in 210 non-pregnant women with active syphilis (defined as RPR ≥ 1:16 without clear classification by duration) in Brazil is comparing cefixime 400 mg twice daily for 10 days (*n* = 140) to a single dose of BPG (*n* = 70) (Table 3) (41); results are expected in 2026.

#### 3.2.4 Efficacy/effectiveness and safety data in pregnant women

##### 3.2.4.1 Treatment for syphilis

Current clinical data on the effectiveness of cefixime for treating syphilis in pregnant women are lacking, and there have been no prospective clinical trials evaluating the dosing, efficacy, or duration of cefixime therapy in pregnant women with syphilis (15, 41). The effectiveness of cefixime to treat fetal infection and prevent congenital syphilis is unknown.

Recognizing the urgent need for alternative options, a phase II, open-label, non-inferiority, randomized controlled trial of oral cefixime (400 mg twice daily for 21 days) vs. intramuscular BPG (2.4 MU × 3 weekly doses) in 230 pregnant women with syphilis is planned (Table 3). This study will assess efficacy in treating syphilis during pregnancy as a primary outcome and will also report the incidence of congenital syphilis, and safety and tolerability.

##### 3.2.4.2 Treatment for other conditions

Cephalosporins, including cefixime, are usually considered safe for use during pregnancy, and cefixime is suggested as an alternative treatment for gonorrhoea during pregnancy (11). Among pregnant women treated with either cefixime (single dose, 400 mg) or ceftriaxone for gonorrhoea, no differences in congenital abnormalities were reported between the two groups (minor abnormalities reported in 7/62, 11%, and 10/60, 17% of infants, respectively) (25). Cefixime has also been used to treat urinary tract infections in pregnancy when given at a dose of 400 mg once-daily for seven days, with no effects on renal function, blood glucose, white blood cells or haemoglobin; however, pregnancy and birth outcomes were not assessed (43).

### 3.3 Amoxicillin

Amoxicillin is an antibiotic from the penicillin class approved for use in pregnancy and used to treat a range of infections. Animal studies have not demonstrated a risk to the fetus, but adequate and well-controlled safety studies in pregnant women are lacking (50). Amoxicillin is sometimes co-administered with probenecid, which inhibits renal tubular secretion of penicillins leading to increased concentrations of these drugs in the blood and prolonged antimicrobial activity. However, it is unclear if co-administration of probenecid is required for successful treatment of syphilis (51). Amoxicillin (or amoxicillin plus probenecid) is not usually considered for the treatment for syphilis in non-pregnant adults (11, 13, 16), but in some countries (e.g., South Africa), it may be offered as an alternative for pregnant women if BPG is unavailable (52). In Japan, where BPG became available and recommended for the treatment of syphilis in 2022, amoxicillin 1,500 mg (500 mg taken orally every eight hours for 4 weeks) without probenecid is offered as an alternative to BPG (53, 54). The duration of therapy varies by syphilis stage: 14–28 days (2–4 weeks) for primary syphilis, 28–56 days (4–8 weeks) for secondary syphilis, and 56–112 days (8–12 weeks) for late symptomatic syphilis during pregnancy (55). European guidelines acknowledge that amoxicillin plus probenecid appears to be effective against *T. pallidum*, but do not specifically recommend it (16).

#### 3.3.1 Preclinical data

Amoxicillin is reported to have *in vitro* activity against *T. pallidum*, with an MIC of 0.02 mg/L (Table 1) (29). Amoxicillin has been shown to cross the placenta at levels sufficient to prevent neonatal group B streptococcal disease, with peak concentrations in cord blood and amniotic fluid estimated to be approximately 18% and 30% of maternal serum levels, respectively (56, 57).

Animal reproductive toxicity studies using amoxicillin were performed in mice and rats at doses up to 2,000 mg/kg (three and six times the 3 g human dose, based on body surface area). These studies showed no evidence of harm to the fetus (Table 2) (50). Effects on testicular, renal, and dental enamel development have been reported in a few animal studies carried out after product licensing (58–60), but no such associations have been observed in humans (see below).

#### 3.3.2 Pharmacokinetics and dosing

In non-pregnant adults, the half-life of amoxicillin is approximately 60 min, and about 60% of the drug is eliminated in the urine within 6–8 h. The usual total daily dose of amoxicillin for treating infections other than syphilis is 750 to 1,750 mg/day given in divided doses every 8 to 12 h, depending on the site and severity of infection. No specific dosing for amoxicillin has been established for treating syphilis during pregnancy.

There are limited PK data on the use of amoxicillin during pregnancy (15). In pregnant women, renal excretion of amoxicillin is estimated to be higher and the half-life shorter (by 19%–31%) than in non-pregnant adults (Table 2) (15, 41). Modelling studies suggest the need for more frequent dosing in later trimesters (15, 61). In one study, a 500 mg oral dose administered at delivery was detected at significantly lower levels in placental and fetal tissue than in maternal tissue, suggesting that higher doses may be required to prevent congenital syphilis (57).

Probenecid is sometimes prescribed with amoxicillin and may lead to a prolonged increase in serum levels of penicillin (15). Probenecid crosses the placenta and is found in cord blood. Animal reproductive toxicity studies have not shown a risk to the fetus, although there are no adequate and well-controlled studies in pregnant women.

#### 3.3.3 Efficacy/effectiveness data in non-pregnant adults with syphilis

Amoxicillin is not considered for treatment of syphilis in non-pregnant adults in WHO guidelines (11), but is recommended in a few countries, for instance Japan (53). A pilot randomized controlled trial comparing amoxicillin (3,000 mg/day) plus probenecid vs. amoxicillin monotherapy (1,500 mg/day) in 112 men with syphilis and HIV reported high serological cure rates at 12 months (94% for amoxicillin plus probenecid, 90% for amoxicillin alone), although non-inferiority of the amoxicillin regimen without probenecid was not achieved (Table 2) (51).

In observational studies, amoxicillin has been shown to be effective in treating syphilis (27). A Japanese observational study found that oral amoxicillin (3,000 mg/day, generally given in doses of 1,000 mg every 8 h, combined with probenecid for 14 days) in adults with HIV achieved a 95% (273/286) serological cure rate at 12 months (62). Another study using amoxicillin (1,500 mg/day, without probenecid, for 28 days) demonstrated a 95% (131/138) cure rate at 12 months in adults with early syphilis (63).

#### 3.3.4 Efficacy/effectiveness and safety data in pregnant women

##### 3.3.4.1 Treatment for syphilis

There have been no clinical trials comparing amoxicillin with BPG in pregnant women with syphilis. Birth outcomes have been reported among 80 women treated for syphilis with amoxicillin (*n* = 66) or ampicillin (*n* = 14) (with or without probenecid) in a retrospective observational study in Japan (55). There were no cases of congenital syphilis reported among 26 pregnancies in women with early syphilis. In contrast, among 45 pregnant women treated for late syphilis with amoxicillin or ampicillin, 15 had infants with congenital syphilis (55). Three women in the study were co-administered probenecid, including one whose infant had congenital syphilis. Two additional case series of pregnancies in women receiving amoxicillin have been published (two women had early syphilis, three late latent syphilis), with no cases of congenital syphilis reported (37, 53).

##### 3.3.4.2 Treatment for other conditions

Although isolated studies of amoxicillin used during pregnancy for other conditions have suggested associations with clinically non-significant neonatal malformations or oral clefts (64, 65), others have reported no increased risk of adverse birth outcomes (66, 67). A systematic review of amoxicillin and congenital anomalies identified four studies, three reporting no association and one (mentioned above 65) reporting an association with oral clefts (67). A broader review of antibiotic safety in pregnancy concluded that amoxicillin, along with other penicillin derivatives, was safe to use during pregnancy (40).

### 3.4 Doxycycline

Doxycycline is a broad-spectrum antibiotic from the tetracycline class. The US FDA does not recommend use in pregnancy primarily based on the potential of the tetracycline drug class to be associated with cosmetic staining of primary dentition in fetuses exposed during the second or third trimester of pregnancy, and concerns about possible enamel hypoplasia and depression of fetal bone growth in animal studies (Table 2) (68). Although there are limited data on the safety of doxycycline itself in pregnancy, several studies have suggested that doxycycline use during pregnancy is not associated with tetracycline class-related adverse effects (69–72). French guidelines note that doxycycline can be used for treating syphilis in the first trimester of pregnancy in cases where desensitization is not possible, and potentially in the second and third trimester if there is a genuine need (73).

Doxycycline is recommended by WHO, US and European guidelines as an alternative to BPG for treatment of syphilis in non-pregnant adults who are unable to take BPG (e.g., due to penicillin allergy, bleeding disorders or lack of drug availability), at a dose of 100 mg twice daily for 14 days for early syphilis or 28–30 days for syphilis of late or unknown duration (11, 13, 16).

#### 3.4.1 Preclinical data

The MIC for doxycycline against syphilis is estimated at 0.10 mg/L (Table 1) (29). Findings from animal studies indicate that tetracyclines cross the placenta, are found in fetal tissues, and can affect skeletal development of the fetus (74). However, there are no published human data specifically demonstrating an association between fetal exposure to doxycycline and cosmetic staining of primary fetal teeth (68). Studies of doxycycline in mice and rabbits carried out between 1967 and 1980 reportedly showed no increase in congenital anomalies with up to six times the maximal human therapeutic dose (3.33 mg/kg) (72). However, an increased frequency of skeletal anomalies associated with maternal toxicity was seen among the offspring of pregnant mice, but not rabbits, treated with 17 times the maximum human dose of doxycycline; in contrast, no teratogenicity was observed at up to 100 times the maximum therapeutic dose in rats and monkeys (72).

#### 3.4.2 Pharmacokinetics and dosing

For adults, the typical dose of doxycycline is 100 mg orally twice daily (or 200 mg once daily) for 14 to 28 days, depending on syphilis disease stage (74). Doxycycline has a relatively long serum half-life of approximately 12–16 h and good bioavailability (15).

Doxycycline PK is expected to be altered by metabolic changes known to occur during pregnancy (such as dilutional reductions in albumin, induction of CYP3A4 metabolism, and increased glomerular filtration), but optimal dosing of doxycycline in pregnancy has not been established (15, 27). Standard adult dosing of doxycycline is currently recommended in pregnancy (15). No studies were found on PK or placental/fetal levels of doxycycline in pregnancy (75).

#### 3.4.3 Efficacy/effectiveness and safety data in non-pregnant adults with syphilis

Several observational studies of doxycycline for treatment of early and late syphilis have shown response rates similar to BPG, ranging from 96% to 100% for early syphilis, 79% for late syphilis, and 65%–75% among people with HIV (27, 76–78). Although there have been no published clinical trials comparing the two drugs, guidelines support the use of doxycycline for treating syphilis in non-pregnant adults who are unable to take penicillin due to allergy or stockouts (11, 13, 16), and a trial is underway in non-pregnant adults (Table 3).

#### 3.4.4 Efficacy/effectiveness and safety data in pregnant women

##### 3.4.4.1 Treatment for syphilis

Historical concerns around the use of first generation tetracyclines in pregnant women have led to a lack of data on doxycycline in pregnant women with syphilis, including for treating and preventing congenital infection (19). In a single case report of a women with syphilis who received doxycycline due to a penicillin allergy, treatment was successful and there were no adverse outcomes (79). A 2025 review concluded that doxycycline could potentially be considered as an alternative for treating syphilis during pregnancy if penicillin or ceftriaxone were unavailable, using standard dosing for non-pregnant adults (15). Pregnant women should be made aware of the potential risks and uncertainties, including around optimal dosing.

##### 3.4.4.2 Treatment for other conditions

Two systematic reviews of doxycycline in pregnancy for indications other than syphilis and an expert review of the Teratogen Information System (TERIS) did not identify any clear evidence of teratogenicity, although all three sources noted a lack of high-quality evidence (72, 74, 80). The expert review concluded that therapeutic doses in pregnancy were unlikely to pose a substantial risk of congenital anomalies, but that data were not sufficient to confirm an absence of risk (74).

In a systematic review from 2016, the authors reported that the safety profile of doxycycline differed significantly from that of tetracycline, with no signs of teratogenicity or tooth staining in infants (72). They reported results of a large retrospective study in Tennessee, in which the rate of major congenital malformations was reported to be 2.5% (among 1,843 infants) following doxycycline use in pregnancy, lower than the background rate for all infants exposed to antibiotics recommended for bioterrorism-related scenarios (2.9%) (81). In a case-control study in Hungary, 0.19% (63/32,804) of healthy control infants were exposed to doxycycline in pregnancy compared with 0.30% (56/18,515) of cases with congenital malformations [odds ratio [OR] 1.6, 95% confidence interval [CI] 1.1–2.3]. When analyses were restricted to the group with doxycycline exposure during organogenesis (second and third months of pregnancy), there were no significant differences between cases and controls for all abnormalities (OR: 1.8, 95% CI: 0.7, 5.0) or for specific birth defects (82).

Another systematic review that included nine studies of doxycycline in pregnancy and over 2,300 women did not find conclusive evidence of a risk of birth defects or miscarriage associated with the use of doxycycline in pregnancy (80). Although one case-control study reporting an association between doxycycline and cardiovascular malformations (OR: 2.4, 95% CI: 1.2–4.7, *n* = 164) was identified (83), this finding was not confirmed in the aforementioned retrospective study from Tennessee, which found no increased risk of cardiac defects in infants exposed to doxycycline *in utero* (relative risk 0.9, 95% CI: 0.5, 1.6, *n* = 1,691) (81). In a separate case-control study, an association between doxycycline and miscarriage was reported (OR: 2.8, 95% CI:s 1.9–4.1, *n* = 36) (84), while in a cohort study of 53 women, lower rates of miscarriage were reported among women treated with doxycycline in the first two trimesters than in untreated women (11% vs. 21%, respectively) (80, 85).

Concerns about tooth staining arise mainly from studies of tetracycline during pregnancy and infancy. Exposure to tetracycline *in utero* causes permanent staining of deciduous teeth only, in approximately 3%–6% of infants, if exposure occurs from around 12 weeks of gestation at the time of tooth calcification. Permanent teeth are not affected as calcification of these teeth occurs after birth (72). Doxycycline has a 2–3 times lower ability to chelate calcium than tetracycline and is therefore less likely to cause tooth discoloration. Although data are limited, two small studies and one randomized controlled trial in children found no evidence of tooth staining associated with use of doxycycline before 8 years of age (when calcification of adult teeth is concluded) (72). Of note, a systematic review on the use of tetracyclines in young children found no evidence of dental staining with doxycycline at any dose in five studies, whereas tetracycline was associated with dental staining in a dose-dependent manner in five of 18 studies (86).

### 3.5 Linezolid

Linezolid is an antibiotic from the oxazolidinone class, generally used to treat serious infections. While preclinical animal studies did not reveal evidence of teratogenicity, embryo-fetal toxicity was observed at maternally toxic doses. The US FDA recommends use in pregnancy if the potential benefit justifies the potential risk to the fetus (87).

WHO supports the cautious use of linezolid in standardized 9-month regimens for treating drug-resistant tuberculosis (TB) during pregnancy, recommending baseline and follow-up laboratory monitoring (haemoglobin, platelets, neutrophils) (88), based on limited evidence of its use in a small number of pregnant women (89, 90). Linezolid is not currently recommended as a treatment for syphilis (Table 1) (12, 13, 16).

#### 3.5.1 Preclinical data

Preclinical studies, including *in vitro* cultivation systems and rabbit models, have demonstrated that linezolid is active against *T. pallidum,* with an MIC of 0.5 mg/L, and has shown efficacy in treating syphilis in animal models (27, 29, 91, 92).

A scoping review on the safety of linezolid in pregnancy found no teratogenic effects and no maternal toxicities in 392 mice, rats, and rabbits given human-equivalent doses in six studies (Table 2) (93). No malformations were seen at maternal exposure levels approximately 6.5 times (mice), equivalent to (rats), or 0.06 times (rabbits) the clinical therapeutic exposure based on the area under the concentration-time curve (AUC) (87).

However, nonteratogenic effects among rat pups were seen at human equivalent doses of 486 mg daily for 12 days based on AUCs, when linezolid was administered to maternal rats from organogenesis through to lactation. Effects included reduced fetal body weight and reduced ossification of sternebrae (often associated with reduced fetal weight) (87, 93). In mice, embryo-fetal toxicities were observed only at doses that caused maternal toxicity (87). A dose 6.5 times the estimated human exposure based on AUCs was associated with increased embryo death, decreased fetal body weights, and an increased incidence of costal cartilage fusion (87). Among 25 mice given a human equivalent dose of 2,189 mg daily for 11 days, eight mice pups experienced total litter loss (93).

#### 3.5.2 Pharmacokinetics and dosing

Linezolid is usually administered at a dose of 600 mg once or twice daily for 10 to 28 days and is used for skin infections, pneumonia and drug-resistant TB (93). Based on its molecular weight and data from animal studies, linezolid is expected to cross the placenta and reach the fetus, but there are no PK studies of linezolid in human pregnancy to confirm this. In a single case report on its use for multidrug-resistant TB in a pregnant woman, maternal plasma concentrations (AUC_0-24_) in the second trimester were 48 mg/L/hour compared with 106 mg/L/hour in the third trimester and 203 mg/L/hour postpartum (94). Dose adjustment is not currently recommended in pregnancy, but may be required (95); PK data in pregnancy are needed (94).

#### 3.5.3 Efficacy/effectiveness data in non-pregnant adults with syphilis

A multicentre randomized controlled trial in men with early syphilis compared oral linezolid (600 mg once daily for five days) to BPG (single intramuscular dose) (91). The trial was stopped early as treatment response was substantially lower in the linezolid group (70%) compared to the BPG group (100%). Although this specific linezolid regimen is not recommended for treating early syphilis, a higher dose for longer duration regimen (600 mg twice daily for 10 days) is now being investigated in a pilot study and a clinical trial (Table 4) (96).

**Table 4:** List of ongoing or planned trials identified through clinical trial registries.

Trial number (ref)	Study title	Phase	Treatment	Country(ies)	N	Start date	End date
CTRI/2024/07/071014	A comparative study of Injection Benzathine Penicillin and Injection Ceftriaxone in the management of Early Syphilis	Phase 2	Ceftriaxone vs BPG	India	60	2024	NR
NCT03660488 (47)	Cefixime for Alternative Syphilis Treatment	Phase 2	Cefixime vs BPG	USA	58	2018	2021
NCT03752112 (41, 45)	Trial Evaluating the Clinical Efficacy of Cefixime for Treatment of Early Syphilis in Non-pregnant Women (CeBra)	Phase 1|2	Cefixime vs BPG	Brazil	210	2019	2026
NCT04958122	Cefixime Clinical Trial	Phase 3	Cefixime vs BPG	USA	400	2021	2027
NCT06877351	Oral Amoxicillin Compared to Penicillin G Benzathine for the Treatment of Acquired Syphilis (AMOXSYP)	Phase 3	Amoxicillin vs BPG	Argentina	120	2024	2027
NCT05309928	Phase 1 Study of the Pharmacokinetics of Amoxicillin in Pregnancy	Phase 1	Amoxicillin	USA	20	2023	2025
JPRN-jRCT1031210615	Efficacy of amoxicillin therapy versus benzathine penicillin G for early syphilis, a multicenter, open-Label RCT	Phase 4	Amoxicillin vs BPG	Japan	208	2022	NR
NCT05980871	Treatment Responses of Early Syphilis to Ceftriaxone Plus Doxycycline	Phase 4	Doxycycline vs Ceftriaxone vs BPG	Taiwan	300	2023	2026
NCT04838717	Clinical Trial of Doxycycline VS BPG for Early Syphilis (SY-DOXY)	Phase 3	Doxycycline vs BPG	France	200	2021	2025
CTRI/2023/05/052508	Comparative study of serological response to doxycycline versus benzathine penicillin in the treatment of early syphilis	NA	Doxycycline vs BPG	India	70	2023	NR
NCT06069141	Comparisons of Treatment Responses of Early Syphilis to Benzathine Penicillin G With or Without Doxycycline	NA	BPG ± doxycycline	Taiwan	688	2024	2026
NCT05548426 (114)	Linezolid for Syphilis Pilot Study	Phase 2	Linezolid 10 days vs Penicillin	USA	24	2023	2026
NCT05069974 (21, 91)	Alternative Antibiotics for Syphilis (Trep-AB, Trep-AB 1200)	Phase 3	Linezolid 600 mg vs BPG	Spain, UK	224	2021	2025

BPG, benzathine penicillin G; NA, not available; NR, not reported; UK, United Kingdom; USA, United States of America; vs, versus.

#### 3.5.4 Efficacy/effectiveness and safety data in pregnant women

##### 3.5.4.1 Treatment for syphilis

There are no data on dosing, efficacy/effectiveness or safety of linezolid in pregnant women with syphilis, and no data on its use for treating fetal infection and preventing congenital syphilis.

##### 3.5.4.2 Treatment for other conditions

Although human safety data for linezolid use during pregnancy are limited, published and postmarketing case reports have not identified a drug-associated risk of major birth defects, miscarriage, or adverse maternal or fetal outcomes (87).

A literature review identified 31 exposed pregnant women (across seven clinical studies and case reports), mostly treated for multidrug-resistant TB (93); no fetal toxicity and no malformations were reported, and no maternal toxicity was reported in 30 women. One case of polyneuropathy (a known potential adverse effect of prolonged linezolid treatment) was reported after long-term use of linezolid (5–6 months) in a pregnant woman (93).

Since the above review, additional pregnancies have been reported from studies in pregnant women with drug-resistant TB who received linezolid (generally 600 mg) as part of their regimen, including 10 in the BeatTB trial (one preterm delivery, no congenital anomalies) (97), four in the PRACTECAL study (three live births, one miscarriage; congenital abnormalities were not assessed) (98), and 41 in the EndTB/STEM-TB studies (99). In the latter study, outcomes were reported for all 43 pregnancies, 41 of which were exposed to linezolid. Among 31 of 43 continuing pregnancies, there were 26 live births, two miscarriages, and four with outcome unknown. Among singleton pregnancies with birth weight reported, seven of 22 (32%) were of low birth weight. One set of twins and seven other babies were born preterm (7/22 singletons, 32%); there was one neonatal death and no congenital abnormalities (99). Overall, no safety concerns were raised in any of these small studies.

### 3.6 Other drugs and/or treatment approaches

#### 3.6.1 Penicillin-based approaches

Other approaches to address issues around shortages of BPG or adherence challenges include the investigation of a single dose of BPG during pregnancy for all stages of syphilis (Table 3) (100). Observational studies in adults with syphilis of late or unknown duration showed similar responses compared with three doses of BPG after adjusting for RPR titre (101, 102). A single subcutaneous infusion of high-dose penicillin (vs. three parenteral doses of BPG) is also being investigated as an option for treating late latent syphilis or syphilis of unknown duration (103). Another trial will assess PK, safety, and tolerability of intramuscular vs. subcutaneous administration of a powdered formulation of BPG in healthy adults (Table 3) (104). A non-inferiority trial of subcutaneous BPG (1 dose of 7.2 MU) vs. intramuscular BPG (2.4 MU × 3 weekly doses) for syphilis during pregnancy is also being planned (Table 3).

#### 3.6.2 Other potential agents

Dalbavancin is a semisynthetic lipoglycopeptide that destabilizes bacterial cell membranes causing cell death (105). It has shown efficacy against *T. pallidum in vitro*. Its low MIC (0.125 mg/L) and long half-life (145 h) mean that a single infusion could potentially produce therapeutic levels for up to six weeks (19, 29). Zoliflodacin, an antibiotic belonging to a new class, the spiropyrimidinetriones, has also shown *in vitro* evidence of activity against *T. pallidum* (27, 29). It acts by inhibiting the bacterial enzyme type II topoisomerase (27). Further studies of both drugs in pregnant and non-pregnant adults are needed.

## 4 Summary

Given limited data from case reports suggesting potential effectiveness for preventing congenital syphilis and a favourable safety profile in human pregnancy when used for other infections, ceftriaxone can be considered as an alternative treatment for syphilis during pregnancy in rare cases where BPG is not available (11). However, while preclinical data and studies in non-pregnant adults support ceftriaxone's safety and efficacy against *T. pallidum*, robust pharmacologic and clinical data on its use for treating syphilis in pregnant women and preventing congenital syphilis remain limited. High quality data on pregnancy and birth outcomes including congenital syphilis would ideally be generated, either in a clinical trial or using surveillance data. The need for daily IM or IV administration of ceftriaxone for 10–14 days is a limiting factor and may be particularly challenging in low- and middle-income settings (15).

**Figure 2:**
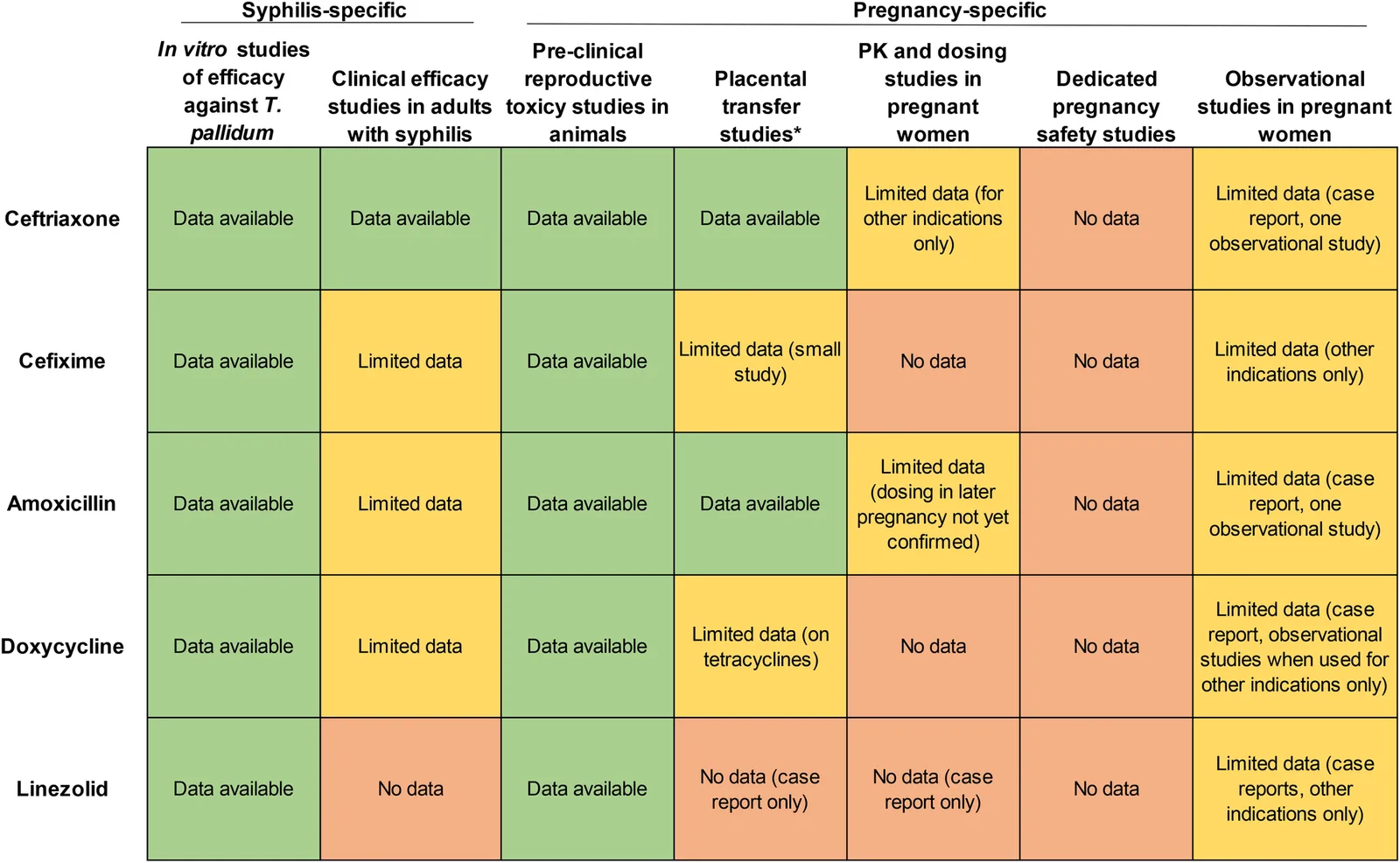
Heatmap showing availability of evidence supporting the use of alternative treatments for syphilis during pregnancy. PK, pharmacokinetic(s); *T. pallidum*, *Treponema pallidum*. *Colours indicate availability of evidence, with green indicating that data are available, yellow indicating that data are limited and/or require confirmation, and orange indicating that no or very limited data are available.

Cefixime has shown promising effectiveness against *T. pallidum* in initial studies in non-pregnant adults and was observed in a small study to cross the placenta. It is considered safe for use in pregnancy for other infections and shows promising effectiveness and a favourable PK profile in non-pregnant adults (Figure 2). However, given the lack of data on efficacy or effectiveness for treating syphilis during pregnancy and for preventing congenital syphilis, cefixime is not currently recommended to treat pregnant women with syphilis. The outcomes of several ongoing studies, including on its efficacy against syphilis in non-pregnant women, on PK in pregnancy, and on safety and efficacy against syphilis in pregnant women will inform its use in pregnant women to prevent congenital syphilis.

Amoxicillin or amoxicillin plus probenecid is not a recommended treatment regimen for syphilis in non-pregnant adults in the United States or Europe (12, 13, 16), but is recommended as an alternative regimen in Japan and other parts of the world (55). Data on its use for treating syphilis during pregnancy and preventing congenital infection are limited, although somewhat encouraging for early syphilis; however, reported cases of congenital syphilis in mothers with late latent syphilis who were treated with this regimen suggest a need for caution (15). Further studies of amoxicillin as an alternative regimen for both early and late syphilis in pregnant women are needed to evaluate the effectiveness (for maternal treatment and prevention of congenital syphilis), optimal dosing and duration, and need for co-administration of probenecid.

Although there are no published clinical trials comparing doxycycline with BPG for treating syphilis, observational studies suggest it is an effective alternative in non-pregnant adults. While animal studies and systematic reviews have not identified any substantive evidence of risk associated with doxycycline use in pregnancy, data are too limited to confirm an absence of risk (Figure 2) (15, 72, 80). Tetracyclines are reported to cross the placenta, but there are no specific data for doxycycline, and its ability to prevent congenital syphilis is not confirmed. There are no PK data on doxycycline in pregnancy, and optimal dosing for treating syphilis during pregnancy has not been established. Although doxycycline is a semi-synthetic derivative of tetracycline, the available limited data do not show an association between doxycycline during pregnancy or early childhood and tooth discoloration, bone growth disturbance or teratogenicity, as has been observed with first generation tetracyclines. However, because of historical concerns about potential tetracycline class effects on fetal teeth and bone, the lack of data on dosing in pregnancy, and lack of evidence on its use for preventing congenital syphilis, the use of doxycycline in pregnant women with syphilis is currently only recommended in limited circumstances, when penicillin and ceftriaxone are not available and with discussion of the potential risks and uncertainties (15).

Preclinical data suggest that linezolid is effective against *T. pallidum*, but confirmation of the dose and duration needed to effectively treat syphilis in non-pregnant adults is needed. There are no data on its use in pregnant women with syphilis, but limited safety data from studies of its use for other infections (primarily drug-resistant TB) in small numbers of pregnant women have not raised specific safety concerns (Figure 2). There is a need for studies of transplacental transfer of linezolid to inform its potential for treating congenital syphilis, and a need for PK and safety studies in pregnant women.

## 5 Discussion

Our review has demonstrated that available data on alternative regimens to BPG for treatment of early or late syphilis during pregnancy to prevent congenital syphilis are not sufficient for a firm recommendation to be made at this stage. Of the five antimicrobials reviewed, ceftriaxone is currently the only one that could potentially be considered as an alternative treatment for syphilis during pregnancy in cases where penicillin is not an option. However, while ceftriaxone, like cefixime, doxycycline and amoxicillin, has been in clinical use for more than three decades, there remains a paucity of data on dosing and PK in pregnancy and a lack of robust pregnancy safety data for all four of these drugs (Figure 2). Our review highlights a critical need for better pregnancy PK and safety data on drugs to treat syphilis during pregnancy.

The need for a more inclusive approach to generating pregnancy safety data has been widely advocated in recent years. Numerous stakeholders have called for fairer inclusion of pregnant and breastfeeding women in clinical trials to enable access to better therapeutic options for prevention and treatment of infections such as HIV, TB, and malaria (106–110). The limited options for treating syphilis during pregnancy, at a time when global shortages of the gold standard treatment, BPG, regularly occur, suggest that novel approaches may also be needed for generating data on preventative and therapeutic agents for syphilis during pregnancy. Approaches proposed for research into treatments for HIV, malaria, and TB include earlier initiation of developmental and reproductive toxicology studies for novel agents, the use of translational science approaches such as physiologically based PK modelling to inform drug dosing in pregnancy, and earlier initiation of PK studies in pregnant women (aligned with the start of phase 3 studies in non-pregnant adults). Once results of developmental toxicity and pregnancy PK studies are available, allowing women who become pregnant in phase 3 trials to remain on the study drug, with appropriate consent and follow-up, provides a cautious route to obtaining early pregnancy safety data (106–108).

Despite the lack of pregnancy PK data across the five drugs included in this review, it is encouraging that several pregnancy PK studies are planned or underway. Results from the IMPAACT 2044 study on the PK and safety of ceftriaxone in pregnant women, expected in 2027, will provide important data to support its use in pregnancy. Additional PK studies of amoxicillin, such as NCT05309928, are also needed to determine the need for dosing adjustment in later pregnancy. An open-label study of amoxicillin PK during pregnancy published since our searches were conducted indicated that 8-hourly dosing is required during pregnancy, with modelling suggesting that a dose of 750 mg would maintain concentrations above the syphilis MIC threshold (111). Although administered orally, the frequent dosing required with amoxicillin (every 8–12 h for 4 or more weeks) can pose adherence challenges. In addition, being a penicillin derivative, amoxicillin is unsuitable for adults with penicillin allergies. While ceftriaxone and amoxicillin may prove to be useful alternatives in specific scenarios, there remains a need for novel, more easily administered treatments for use in pregnant women, particularly those with penicillin allergy.

Cefixime and linezolid have emerged more recently as candidates for treating syphilis in non-pregnant adults. Should clinical trials demonstrate non-inferiority compared with BPG, data on their ability to cross the placenta and thereby prevent congenital syphilis and treat fetal infection will be required. It is encouraging that pregnancy PK and placental transfer studies of cefixime are planned, with results expected in 2026–2027. Similar studies of linezolid are also needed, along with continued evaluation of linezolid's safety in pregnancy. For both antibiotics, questions remain around which data are needed by policy makers to provide recommendations on their use in pregnant women with syphilis. For antiretrovirals, demonstrated therapeutic efficacy in non-pregnant adults with HIV is considered sufficient to infer efficacy in pregnancy—both for maternal treatment and for prevention of vertical transmission—if therapeutic drug levels are maintained in pregnancy (110). Whether the same holds true for antimicrobials used to treat both maternal and fetal syphilis infection *in utero* requires careful consideration. Evidence of efficacy or effectiveness in non-pregnant adults combined with pregnancy PK data and data on placental transfer and short-term safety may be sufficient to support the use of these drugs in pregnant women when BPG is not available or cannot be used, provided that robust post-marketing and/or post-implementation surveillance studies or pregnancy registries are established to monitor pregnancy and infant outcomes prospectively (110, 112, 113).

Questions around the potential use of doxycycline for treating syphilis during pregnancy require a different approach. There is a need for pregnancy PK data on doxycycline, but historical concerns about the tetracycline class have hindered the generation of such data. With trials of doxycycline for treatment of syphilis in adults currently underway (Table 4), establishing PK in pregnant women would be timely. In parallel, existing sources of data on exposure to doxycycline in pregnancy, including when used for indications other than syphilis, could provide additional evidence on its safety in pregnancy. Given the limited therapeutic landscape for pregnant women with syphilis and long delays in identifying new candidate drugs, repurposing of other existing antibiotics used for other indications should be explored where possible.

Macrolides such as azithromycin and erythromycin have historically been used for treating syphilis, but emerging macrolide resistance (reaching 90% in some regions) is likely to limit their use (15–19). WHO still currently suggests erythromycin for pregnant women for rare situations where BPG cannot be used, but notes that it has limited placental passage and that the newborn must therefore be treated with penicillin (9, 20). This recommendation is likely to be reviewed considering the emerging drug resistance.

Although this review focused on potential alternatives to penicillin for situations where BPG is not available or cannot be administered, there is also a need for therapeutics (ideally administered orally) that could be offered as an alternative choice to BPG, for example for pregnant women who decline injections or who are unwilling to undergo penicillin desensitization. High quality evidence demonstrating safety and non-inferiority to BPG for treating syphilis in non-pregnant adults and an understanding of effectiveness in preventing congenital syphilis would be needed before such options could be recommended as alternative choices. In the meantime, concerted efforts are needed to address global BPG shortages and disruptions to supply chains (18) to avoid preventable cases of congenital syphilis and perinatal deaths due to untreated maternal infection.

While BPG remains the standard of care for treating syphilis during pregnancy and preventing and treating congenital syphilis *in utero*, there have been promising developments around the use of alternative drugs for syphilis in non-pregnant adults. This review has highlighted a critical need for better pregnancy PK and safety data on medicines to treat syphilis during pregnancy, including studies of placental and fetal transfer given the need to prevent congenital syphilis. Direct evaluation of efficacy and effectiveness in pregnancy, in carefully designed studies emphasizing safety and ethical standards, should be considered wherever possible. Evaluation of new as well as repurposed drugs for treatment of syphilis should involve a pregnancy investigation plan, with generation of high-quality pregnancy data to help inform and optimise the care of pregnant women with syphilis and prevent congenital syphilis.
